# Ultrasound‐Based Local Lung Motion Assessment Using Synthetic Lateral Phase

**DOI:** 10.1002/jcu.23908

**Published:** 2025-01-25

**Authors:** Christopher M. Fung, Jonathan M. Rubin, Jing Gao, James D. Hamilton

**Affiliations:** ^1^ Department of Emergency Medicine University of Michigan Ann Arbor Michigan USA; ^2^ Department of Radiology University of Michigan Ann Arbor Michigan USA; ^3^ Department of Clinical Sciences Rocky Vista University Billings Montana USA; ^4^ JD Hamilton Consulting Brighton Michigan USA

## Abstract

**Background:**

Ultrasound lung surface motion measurement is valuable for the evaluation of a variety of diseases. Speckle tracking or Doppler‐based techniques are limited by the loss of visualization as a tracked point moves under ribs or is dependent.

**Methods:**

We developed a synthetic lateral phase‐based algorithm for tracking lung motion to overcome these limitations. To validate the technique, we generated simulated lung motion images. We also obtained lung ultrasound cines from a healthy volunteer and a mechanically ventilated COVID‐19 patient. In the healthy volunteer, the respiratory pattern varied between breath‐hold, regular, and rapid shallow breathing.

**Results:**

The measured displacement was within 3% of the ground truth for simulated cines. In both the healthy volunteer and COVID‐19 patients, measured displacement was greatest in the lower and lateral zones of the lung when the ipsilateral side was compared. In the healthy volunteer, when the respiratory pattern was varied, measured displacement was greater in regular breathing compared to rapid shallow breathing and compared to breath‐hold patterns in both the upper and lower lung zones.

**Conclusion:**

Estimation of lung surface displacement using a synthetic lateral phase‐based approach is feasible. Future human studies should validate this approach against a direct measurement of lung surface movement.

## Graphical abstract

Representative workflow images from lung motion tracking software. (A) Screen capture of user marking lung surface. (B) Screen capture of displacement heatmap and selection of region of interest for motion tracking. (C) Video of motion tracking. (D) Screen capture of breath marking.

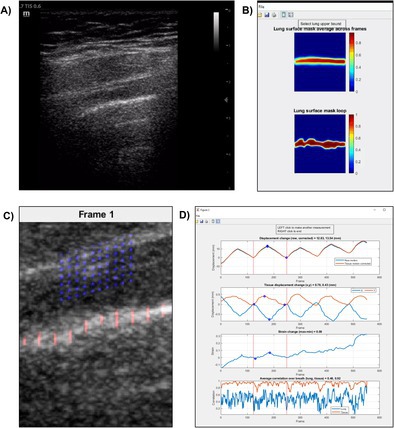

## 1 Introduction

Diseases of the lung often affect it non‐uniformly resulting in regional differences in lung physiology [1, 2]. Current techniques for evaluation of lung physiology such as pulmonary function testing and spirometry are global in nature and do not inform clinicians about regional lung function. Functional and mechanical properties of different regions of the lungs are not uniform because lung tissue experiences cyclic mechanical strain and shear distortions due to asymmetric deformations and surface tensions at air‐tissue interfaces [3, 4]. Multiple factors could impact regional lung parenchyma structure and tissue deformation, such as asymmetry of the chest cavity and rigidity of the mediastinum, which predispose to nonuniform distributions of mechanical stress, and lobar expansion [5]. In addition, some lung diseases (idiopathic pulmonary fibrosis, asymmetrical interstitial lung disease) exhibit heterogeneous pathologic changes in the lung [6]. Real‐time, dynamic assessment of regional lung function would greatly aid clinicians in both the diagnosis and management of lung disease.

Standard radiographic techniques, such as computed tomography (CT) evaluate only static anatomy as a proxy for function. Several technologies have emerged to address this clinical need. CT of the chest can evaluate lung function by obtaining a series of static images during the respiratory cycle or via changing patient positioning. Regional lung movement and tissue deformation can be estimated via image segmentation and software to calculate the movement of different segments of the lung. The fidelity of CT can also be augmented via the addition of inhaled noble gas contrast which has shown similar performance to pulmonary function testing when measuring lung ventilation [3]. Magnetic resonance imaging (MRI)‐based techniques making use of hyperpolarized inhaled gas to magnify the MRI signals from air‐containing lungs have been shown to provide global pulmonary function data similar to spirometry while retaining regional information [7]. However, MRI is limited by significant cost and access issues. Other technologies such as electrical impedance (EIT) tomography and nuclear medicine‐based ventilation imaging have long been used to image local lung ventilation but create images with inferior resolution to CT and MRI [7].

The clinical use of ultrasound to image the lung has dramatically increased in the last two decades with applications across a multitude of diseases and specialties of medicine [8]. However, the use of lung ultrasound has generally been limited as a bedside tool for diagnosis rather than a real‐time monitoring which requires both independence from a trained sonographer and the capability to track lung motion. Color and power Doppler sonography as an adjunct to B‐ and M‐mode imaging for the detection of pneumothorax has been described as the confirmation of the presence or absence of lung sliding (motion along lung‐pleura interface) detected with B‐mode sonography [9]. Unfortunately, color and power Doppler assessments of lung surface motion are not quantitative and are angle‐dependent. Tissue movement, such as adjacent cardiac or vascular pulsations, as well as hand movement of the sonographer may affect the accuracy of color and power Doppler in assessing lung surface motion [9].

Ultrasound lung surface wave elastography (ULSWE), both ex vivo [10] and in vivo [11, 12, 13], has been used to interrogate the mechanical properties of the lung surface. Lung function, pulmonary edema [13], and interstitial lung disease [11, 12] can be evaluated using noninvasive ULSWE, which may be adjunct to high‐resolution CT for the noninvasive evaluation of interstitial lung disease with sensitivity and specificity of 92% and 89% for differentiating between healthy lungs and interstitial lung diseases [12]. However, ULSWE requires an operator to place two devices, an oscillator to create harmonic vibration and an ultrasound transducer, simultaneously in the same intercostal space and the lung surface must be stationary so the real‐time function cannot be assessed. The use of speckle tracking to measure strain and displacement of the lung surface during breathing has been demonstrated in a mouse model of pulmonary fibrosis as well as in healthy human volunteers [14, 15]. However, a major limitation of speckle tracking in lung imaging is that tracking becomes impossible if the tracked surface moves under a rib, which effectively blocks the sound from reaching the lung surface. Additionally, strong specular reflections from the lung surface reduce the quality of the speckle pattern for tracking, resulting in correlation peak hopping for speckle‐tracking methods that utilize cross‐correlation or other image‐matching‐based tracking methods [16]. Therefore, it is necessary that any robust lung motion assessment using ultrasound employ a tracking method that does not follow specific speckle spots and is robust to the ultrasound signal characteristics of the lung surface. The aim of our study was to detail the development of a synthetic lateral phase technique for tracking lung motion that overcomes many of the limitations of the previously described ultrasound techniques and demonstrate its use in a healthy human volunteer and a mechanically ventilated patient with significant lung disease.

## 2 Materials and Methods

### 2.1 Study Design

This was a technical development study utilizing prospectively obtained lung ultrasound cine loops from a healthy volunteer and a mechanically ventilated patient with COVID‐19‐associated respiratory failure. The study was approved by the IRB of the University of Michigan (HUM00079685 and HUM00192125). We also performed a computer simulation of lung motion where the simulated data is a horizontal strip of laterally moving ultrasound speckles that represents the translating lung surface.

### 2.2 Image Acquisition

Lung ultrasound cine loops were obtained using a standardized image acquisition protocol that is similar to routine lung ultrasound performed in clinical settings [8]. Briefly, the chest wall was divided into upper and lower zones and scanning was performed in each of four zones on both the right and left hemithorax. A total of eight zones were imaged per participant. For the healthy volunteer, 6 s cine loops were acquired in breath‐hold, rapid breathing (approximately 40–50 breaths/min), and regular breathing (approximately 12 breaths per minute). Scanning was performed in the coronal or sagittal orientations with a 3–13 MHz linear transducer (Mindray L12‐4) on a Mindray M9 ultrasound system (Mindray North America, Mahwah, NJ) with the following settings: focal plane set to the pleura line, maximum depth at three to four times the distance from the skin surface to the pleural line, frame averaging turned off, and spatial compounding off. All images were exported as uncompressed DICOM files for analysis.

### 2.3 Lung Motion Tracking Algorithm

A detailed description of the lung motion tracking algorithm and component steps is available in Appendix S1. Briefly, our tracking method utilizes a synthetic lateral phase [17] to measure local lung motion at pixel locations through a B‐mode cine loop. In this approach, B‐mode images are one dimension spatially filtered along a dimension aligned with the lung surface using a complex, finite impulse response (FIR) bandpass filter that selects frequencies on one side of DC, like a Hilbert transform. The resulting complex images are analogous to RF ultrasound signals, with phase proportional to position in the image (i.e., distance) and the center spatial frequency. The interframe displacement at a pixel of interest is calculated from the phase change between subsequent frames for a sub‐image (kernel) about the pixel. The phase value is converted to pixels of motion by dividing the result by the phase of the first lag of the reference kernel autocorrelation. The process is repeated for all pixels of the lung surface and image pairs of the B‐mode CINE loop. The lung displacement through a breath cycle for a given pixel is measured by integrating the interframe displacements (i.e., velocities) through the image loop, similar to tissue Doppler‐based displacement measurements.

To calculate strain, the tracked lung surface is divided into two equal‐length segments. The time‐dependent displacement of each segment is subtracted and normalized by the distance between the weighted center of valid pixels for each segment. Details of the strain calculation are presented in Appendix S1.

### 2.4 Generation of Simulated Data

**FIGURE 1:**
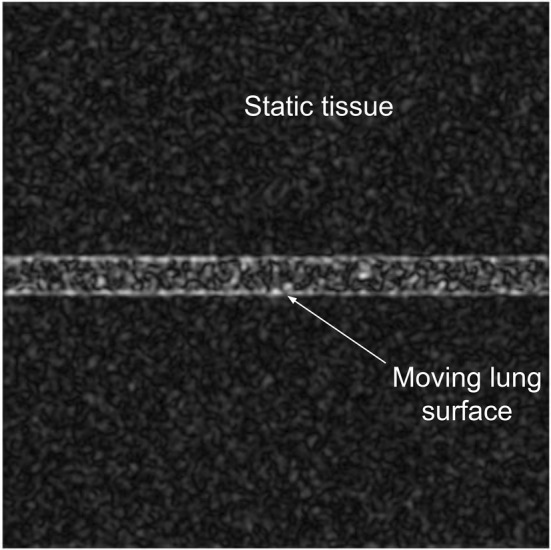
Representative B‐mode image of simulated lung surface. Two scatterer objects were used to create the image. One set of scatterers generated the lung surface, represented by the horizontal strip in the center of the image. The scatters for the lung surface have both strain and displacement parameters assigned to simulate lung motion. The second scatter object creates the background, a static speckle with no motion or strain.

The accuracy and robustness of the tracking system were quantified using simulated ultrasound B‐mode image data representing lung surface motion and deformation. Briefly, the simulated data is generated by randomly distributing scatters on a spatial grid and applying weighting to each scatterer based on the desired acoustic backscatter strength. To represent lung images, a horizontal strip of scatterers with high amplitude weighting compared to stationary background scatterers was generated. The strain and displacement parameters defined in the software determine the position of each scatter for each frame. B‐mode images are created by convolving the scatter distributions with a simulated RF point spread function (PSF) followed by basebanding and magnitude detection. An example simulated B‐mode image is shown in Figure 1.

### 2.5 Data and Statistical Analysis

Three independent reviewers (C.M.F., J.M.R., J.D.H.) viewed each cine loop and identified a region of interest for motion tracking. After applying the motion tracking software, each reviewer marked the borders of each tracked respiratory cycle (full inspiration and expiration) to calculate displacement. Tracking videos were created from each cine clip and were viewed by each reviewer to assess the quality of lung surface tracking. Average displacement was calculated from all breaths marked by the reviewer from each cine loop. A representative animation of the user image processing workflow is depicted in the graphical abstract and animated here (https://docs.google.com/presentation/d/1_mVWTNDVwdfMKpX3E_Av9UQJE6rV_j9F/). Descriptive statistics were calculated for average displacement in the lateral (horizontal lung surface movement) and near field to deep dimensions (vertical tissue movement). Inter‐reviewer reliability was assessed between the three reviewers using a two‐way random effects intraclass correlation coefficient for agreement. To assess lung surface movement, we compared displacement between anterior and lateral lung zones as well as upper versus lower lung zones on each side of the thorax. In the healthy volunteer, we also compared measured displacement across respiratory patterns. A two‐sample *T*‐test and limit of *p* < 0.05 was used to assess the difference in average displacements. Data preparation and all statistical analyses were conducted using R (v4.2.1) in R Studio (v2022.2.3). Data visualization was conducted in Tableau (Tableau Software).

## 3 Results

### 3.1 Simulated Lung Surface Tracking

To evaluate the accuracy of the lung tracking software, simulated image data was processed by the tracking algorithms and the strain and displacement measurements were compared with the known simulated strain and displacement values used to generate the simulated data. In this case, simulated B‐mode loops representing lung motion with a peak strain and displacement of 9.5% and 36 pixels, respectively, were generated. These values approximate strain and displacement values observed in the mid to lower portions of a healthy lung with normal breathing patterns. Ten image loops with different speckle realizations were generated. Measurements of peak‐to‐valley strain and lateral displacement were performed by a single observer using the tracking software. A summary of the results is shown in Table 1. The average measured value for both strain and displacement is within 3% of the true value. Although the simulated image data is simplified compared to clinical lung images, the validation analysis demonstrates the potential accuracy and repeatability of the technique.

**TABLE 1:** Intraclass correlation coefficient for measures of displacement.

Measurement	ICC^a^ (95% CI)
Lung displacement (lateral dimension)	0.98 (0.97–0.99)
Lung displacement corrected for tissue displacement	0.98 (0.97–0.99)
Tissue displacement (near field to deep field dimension)	0.98 (0.97–0.99)

^a^ Two‐way random effects model for agreement.

### 3.2 Inter‐Reviewer Reliability of Lung Surface Measurements

Using the 24 cine loops obtained from the healthy volunteer across all breathing conditions and 8 cine loops obtained from a patient with COVID‐19 pneumonia, we estimated the intraclass correlation coefficient with three reviewers for average displacement. All observed breaths from each cine loop were measured and the intraclass correlation coefficients (ICC) are reported in Table 2. ICC was > 0.9 for measured displacement of the lung surface (lateral movement), tissue displacement, and lung surface displacement corrected for tissue displacement.

**TABLE 2:** Measured motion parameters from simulated B‐mode image loop compared with truth.

Motion parameter	Average of measurements	Standard of measurements	Truth
Displacement	37.2 pixels	1.2 pixels	38 pixels
Strain	9.52%	0.71%	9.5%

### 3.3 Lung Surface Tracking in a Healthy Volunteer

**FIGURE 2:**
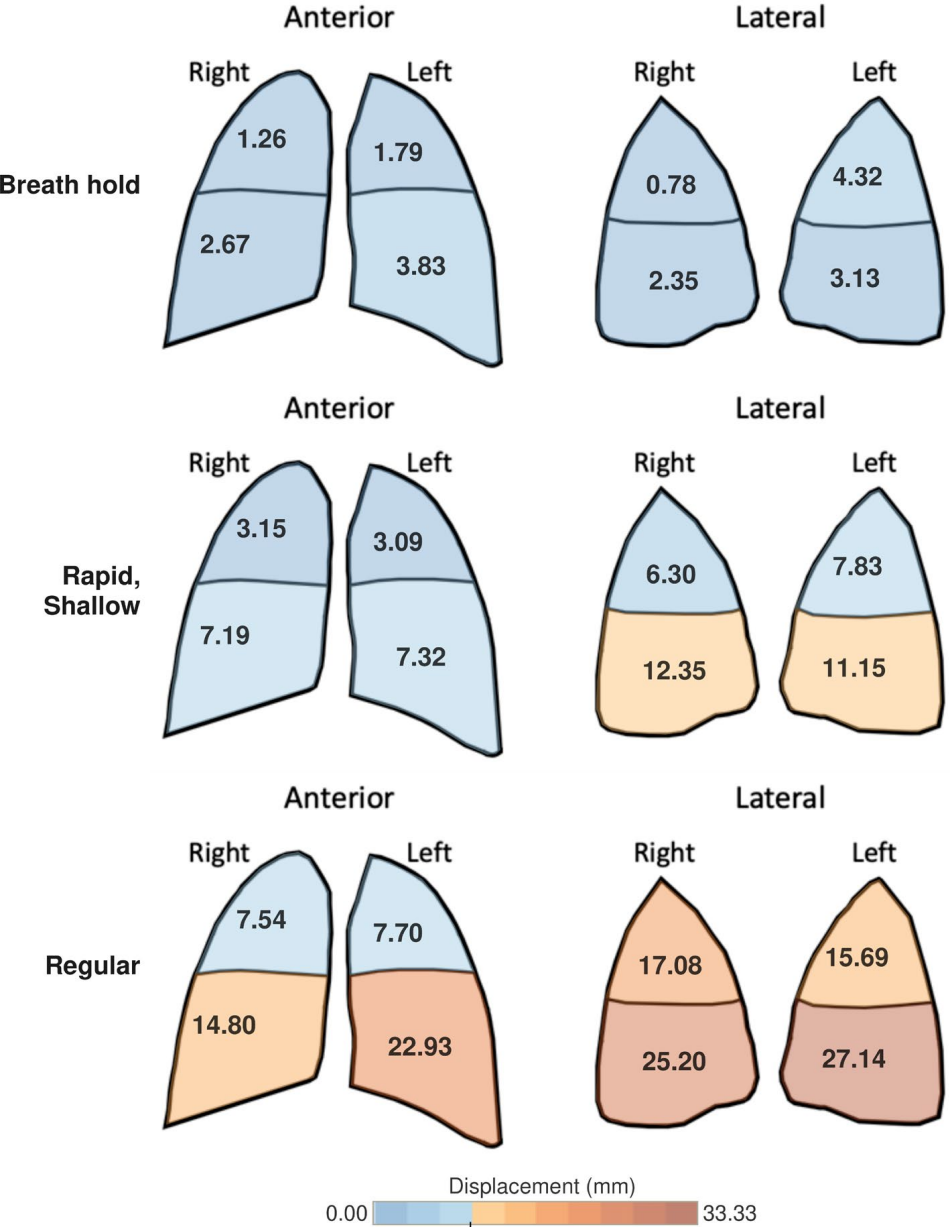
Heat map representing the vertical displacement in each of four (anterior and lateral) lung zones in three breathing conditions for a healthy volunteer. Average displacement is reported in millimeters for each zone. Average displacement was greater in the lower lung zones and regular breathing when compared to breath‐hold or rapid, shallow breathing.

Images were obtained in breath‐hold, normal breathing, and rapid, shallow breathing from a healthy volunteer with no known lung disease. The average displacement in each of the imaged lung zones for each breathing condition is represented in Figure 2. Displacement was greater in the lower zones when compared to ipsilateral upper zones (Table 3). Similarly, displacement was greater in the lateral lung zones when compared to the ipsilateral anterior zone (Table 4). When compared across breathing patterns stratified by upper and lower lung zones, regular breathing demonstrated significantly more lung surface displacement than rapid, shallow breathing and breath‐hold conditions (Table 5).

**TABLE 3:** Lung surface displacement in healthy volunteer—lower compared to upper zones.

Breathing pattern	Location	Mean displacement (mm)		Mean difference (95% CI)	*p*
		Upper zone	Lower zone		
Breath‐hold	Right anterior	1.26	2.67	1.41 (0.72–2.10)	< 0.001
	Right lateral	0.78	2.35	1.57 (−1.77 to 4.91)	0.33
	Left anterior	1.79	3.83	2.04 (1.45–2.63)	< 0.001
	Left lateral	4.32	3.13	−1.19 (−3.13 to 0.75)	0.21
Rapid, shallow	Right anterior	3.15	7.19	4.04 (3.33–4.75)	< 0.001
	Right lateral	6.3	12.35	6.05 (5.24–6.86)	< 0.001
	Left anterior	3.09	7.32	4.23 (3.21–5.25)	< 0.001
	Left lateral	7.83	11.15	3.32 (1.81–4.83)	< 0.001
Regular	Right anterior	7.54	14.8	7.26 (5.61–8.91)	< 0.001
	Right lateral	17.08	25.2	8.12 (5.66–10.58)	< 0.001
	Left anterior	7.70	22.93	15.23 (12.13–18.33)	< 0.001
	Left lateral	15.69	27.14	11.45 (6.76–16.14)	< 0.001

**TABLE 4:** Lung surface displacement in healthy volunteer—lateral compared to anterior zones.

Breathing pattern	Location	Mean displacement		Mean difference (95% CI)	*p*
		Anterior zone	Lateral zone		
Breath‐hold	Right upper	1.26	0.78	−0.45 (−0.79 to 0.17)	0.005
	Right lower	2.67	2.35	−0.32 (−3.72 to 3.08)	0.84
	Left upper	1.79	4.32	2.53 (1.86–3.20)	< 0.001
	Left lower	3.83	3.13	−0.70 (−2.61 to 1.21)	0.45
Rapid, shallow	Right upper	3.15	6.3	3.15 (2.70–3.60)	< 0.001
	Right lower	7.19	12.35	5.16 (4.18–6.14)	< 0.001
	Left upper	3.09	7.83	4.74 (3.73–5.75)	< 0.001
	Left lower	7.32	11.15	3.83 (2.31–5.35)	< 0.001
Regular	Right upper	7.54	17.08	9.54 (8.00–11.08)	< 0.001
	Right lower	14.8	25.2	10.4 (7.87–12.93)	< 0.001
	Left upper	7.7	15.69	7.99 (6.40–9.58)	< 0.001
	Left lower	22.93	27.14	4.21 (−1.19 to 9.61)	0.12

**TABLE 5:** Lung surface displacement in healthy volunteer—compared by breathing pattern.

Breathing pattern	Location	Mean displacement (mm)	Mean difference (95% CI)	*p*
Breath‐hold	Upper zones	2.04	Ref.	
Rapid, shallow	Upper zones	5.10	3.06 (2.18–3.94)	< 0.001
Regular	Upper zones	12.00	9.96 (8.32–11.60)	< 0.001
Breath‐hold	Lower zones	2.99	Ref.	
Rapid, shallow	Lower zones	9.50	6.51 (5.26–7.76)	< 0.001
Regular	Lower zones	22.52	19.53 (17.27–21.79)	< 0.001

### 3.4 Lung Surface Tracking in a Mechanically Ventilated Patient With COVID‐19 Pneumonia

**FIGURE 3:**
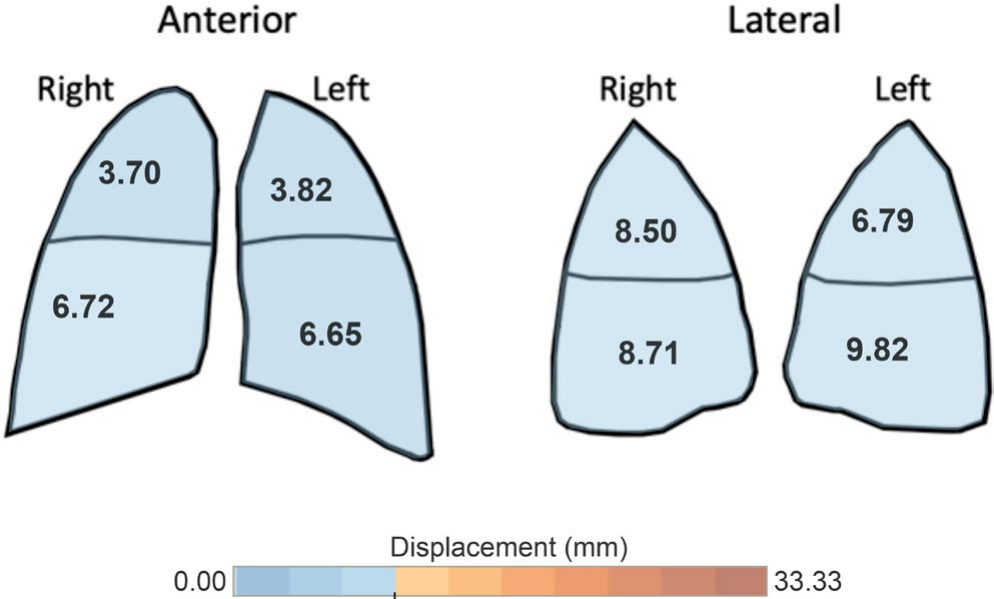
Heat map representing the vertical displacement in each of four (anterior and lateral) lung zones during two observations 3 days apart in a mechanically ventilated patient with COVID‐19 pneumonia. Average displacement is reported in millimeters for each zone. Average displacement was greater in the lower lung zones and in observation 2 when compared to observation 1. The right lateral lung zone was not accessible for imaging at observation 2.

**FIGURE 4:**
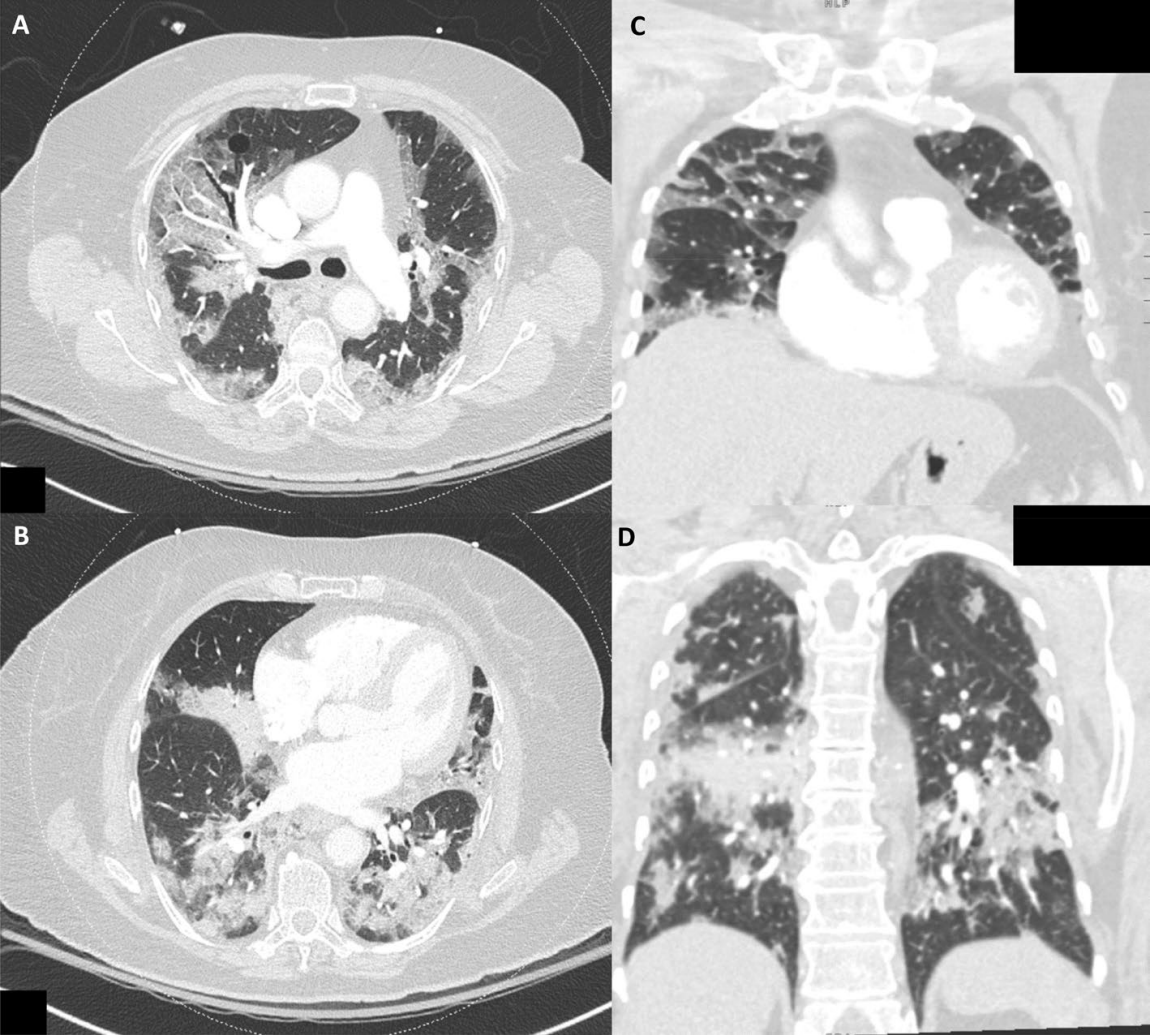
Computed tomography images of the chest from the COVID‐19 patient who received lung ultrasonography to track lung surface movement on the same day. (A) Axial image of the upper lungs at the approximate level corresponding to upper lung ultrasound zones. (B) Axial image of the lower lungs corresponding to the lower lung ultrasound zones. (C) Coronal image of the anterior lungs. (D) Coronal image of the posterior lungs.

Images were obtained from a patient who was receiving mechanical ventilation for COVID‐19 pneumonia and acute hypoxic respiratory failure. Average displacement in each imaged lung zone is displayed in Figure 3 and representative corresponding CT images from the same day are displayed in Figure 4. Lateral and lower lung zones had higher average displacement when compared to ipsilateral anterior and upper lung zones (Table 6).

**TABLE 6:** Lung surface displacement in COVID‐19 pneumonia patient.

Location	Mean displacement (mm)		Mean difference (95% CI)	*p*
	Upper zone	Lower zone		
Right anterior	3.7	6.72	3.02 (2.37–3.67)	< 0.001
Right lateral	8.5	8.71	0.21 (−0.77 to 1.19)	0.66
Left anterior	3.82	6.65	2.83 (1.10–4.56)	0.003
Left lateral	6.79	9.82	3.03 (2.30–3.76)	< 0.001

Location	Mean displacement (mm)		Mean difference (95% CI)	*p*
	Anterior zone	Lateral zone		
Right upper	3.7	8.5	4.80 (3.98–5.62)	< 0.001
Right lower	6.72	8.71	1.99 (1.30–2.68)	< 0.001
Left upper	3.82	6.79	2.97 (1.90–4.04)	< 0.001
Left lower	6.65	9.82	3.17 (1.83–4.51)	< 0.001

## 4 Discussion

In this technical demonstration, we have shown in both computer simulation and two human cases that ultrasound motion estimation of the lung surface using synthetic lateral phase can estimate the displacement of the lung surface. In a healthy volunteer across varying breathing patterns, measurements using our novel approach corresponded to the greater intrinsic displacement of the dependent portions of the lung as well as the increased displacement expected from deeper breathing. Likewise, in a mechanically ventilated patient despite multifocal pneumonia due to COVID‐19, we measured a similar increase in lung displacement in the lower and lateral lung zones. The inter‐reviewer reliability of our measurements was excellent (ICC > 0.9) for both lung and tissue displacement. Overall, the synthetic lateral phase approach to lung surface motion estimation was reproducible across users and physiologic conditions.

Traditional speckle tracking approaches, like those used in echocardiography, integrate the motion of the tissue through the acquired image loop (i.e., ‘follows’ the tissue to quantify motion) and cannot accommodate the loss of speckles in the image due to rib shadows. Our technique measures motion at fixed locations (i.e., image pixels) like tissue Doppler, and does not require tracking tissue regions through the breath cycle. This makes the technique robust to rib shadowing and simply compares the phase of regions of successive images to determine motion, avoiding the problem of peak hopping associated with block‐matching tracking algorithms operating on poor image quality data. An added benefit is this phase‐based method calculates interframe motion with sub‐pixel precision and does not require additional processing like phase zero‐crossing or correlation function peak interpolation that is needed for cross‐correlation‐based speckle tracking sub‐pixel measurements. Our method provides accurate local, instantaneous measurements of lung surface motion.

However, synthetic lateral phase approaches are not without their own technical limitations. Like Doppler techniques, aliasing can occur for large displacements that produce greater than 180° phase change between frames, resulting in incorrect inter‐frame motion measurements. Additionally, the accuracy of the motion measurement depends on the center frequency of the synthetic lateral phase data which can be affected by the spatial frequency distribution of the images. The approach is best suited for tracking motion in one dimension since simultaneous tracking in two dimensions requires two sets of lateral phase images and additional computations to distinguish phase changes associated with motion in each dimension [17]. Our method was applied to 2D B‐mode image loops. Although the acquisition geometry was adjusted to maximize the in‐plane motion of the lung surface, any out‐of‐plane motion was not measured. Additionally, in our pilot study, we constrained our comparisons to known physiologic variation in lung displacement—where the dependent lateral and lower lung zones move more during each breath and deeper breaths produce more lung surface movement. Future studies should utilize a gold standard for measuring lung surface movement, such as cross‐sectional imaging in maximal inspiration and expiration, paired with lung ultrasound to determine the accuracy of lung motion detection. Our workflow was also limited by the need for manual region of interest selection of the lung surface and identification of breaths in displacement plots. However, recent advances in automated feature detection could easily be combined with our approach to fully automate the process [18]. Finally, it is important to note that breath‐hold displacements are not zero. All our measurements are absolute (peak‐to‐valley) displacements, therefore, any variations in the estimates have to be greater than zero. Any errors in the motion estimates or induced motion in the lungs, such as displacements due to cardiac or adjacent vessel wall motions, will add positive values to the lung surface motion estimates. Thus, motions during breath holding are to be expected, and our analyses show that these motions are small and significantly less than motions produced when true breaths are taken.

Lung surface motion tracking has a multitude of potential applications and advantages over current techniques that provide clinicians with only static or global information regarding lung ventilation. In our study, we chose to evaluate the performance of our technique in a mechanically ventilated patient for purposes of demonstration due to the potential applications in measurement of regional lung ventilation to provide real‐time feedback to critical care physicians, or even to the ventilator directly, regarding changes in disease state or efficacy of applied changes to ventilator settings. For example, continuous tracking of lung motion could be used not only to monitor for improvement (or failure to improve) in lung ventilation due to a change in inspiratory pressure but also to detect life‐threatening events such as the development of a pneumothorax prior to clinical signs of decompensation.

## 5 Conclusion

We have demonstrated that a synthetic lateral phase technique for lung surface motion tracking is feasible in healthy and diseased human lungs. Our results show that regional differences in measured lung surface displacement correspond to expected patterns of lung motion where more dependent areas of the lung move more. Breathing patterns with smaller breaths also demonstrated less lung surface displacement. Future development should optimize motion tracking to evaluate lung surface strain.

## Author Contributions

C.F., J.R., J.G., and J.H. were the primary writers of the manuscript. J.R. and J.H. conceptualized the study. J.H. was the primary software engineer and was primarily responsive to algorithm development. C.F. was the primary data analyst. All listed authors contributed substantially to the design of this study. All listed authors participated in editing the manuscript. C.F. supervised all aspects of study design, regulatory approval, data collection, analysis, and manuscript preparation.

## Conflicts of Interest

The authors declare no conflicts of interest.

## Supporting information


**Data S1.** Supporting Information.

## Data Availability

The data that support the findings of this study are available on request from the corresponding author.
