# Supplementary material for: Ultrasound‐Based Local Lung Motion Assessment Using Synthetic Lateral Phase
Source: J Clin Ultrasound. 2025 Jan 25;53(4):639–46. doi: 10.1002/jcu.23908 (PMC12087715; doi:10.1002/jcu.23908)
Supplement: Supplementary file 1 — Data S1. Supporting Information. [file JCU-53-639-s001.docx]

# **Methods Supplement**


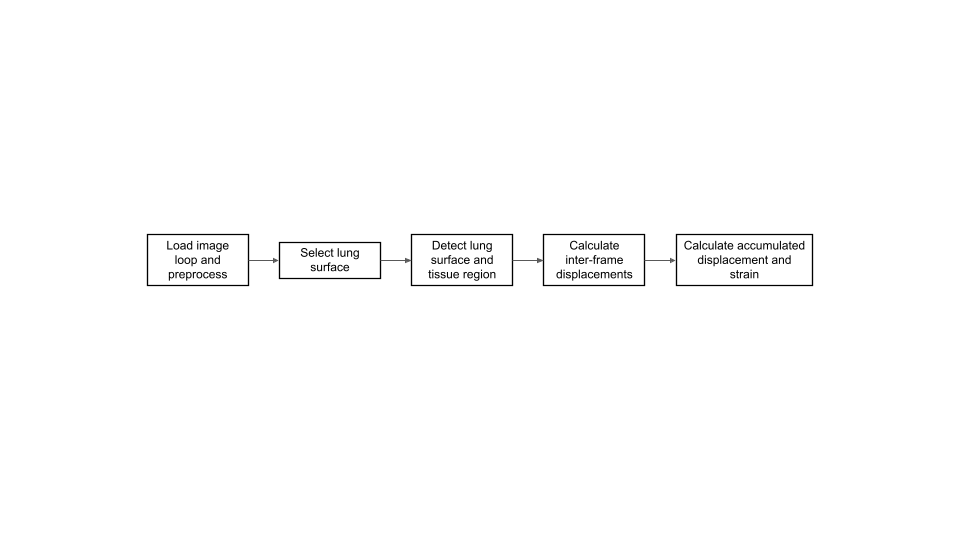


Supplemental Figure S1: Overview of lung tracking method.

##

## *Method Overview*

An overview of the lung tracking method is shown in Supplemental Figure S1 and details for each stage of the algorithm are presented in the sections below. The input data is a B-mode DICOM image loop. Our method is independent of scanner type or manufacturer and runs off-line on a personal computer or workstation. The software was developed using Matlab^TM^ (Mathworks, Natick, MA). The initial step of the method requires the user to select a DICOM image loop from which the software extracts image frames and DICOM fields (e.g., pixel size, frame rate, etc.). The image data is preprocessed (e.g., filtered) to prepare the data for the operations of the subsequent stages. The user is then prompted to select the lung surface by identifying two end points of a line that approximately overlays the lung surface. Subset images are extracted from the full B-mode image loop based on the line position and processed to identify pixels of the lung surface as well as surrounding tissue directly above the lung surface (between ultrasound probe and lung surface). Next, the inter-frame displacement of the lung surface and tissue regions are measured using the synthetic phase approach described below. Finally, the accumulated displacement of the lung surface and surrounding tissue are calculated along with the strain (stretch and relaxation) of the lung surface.


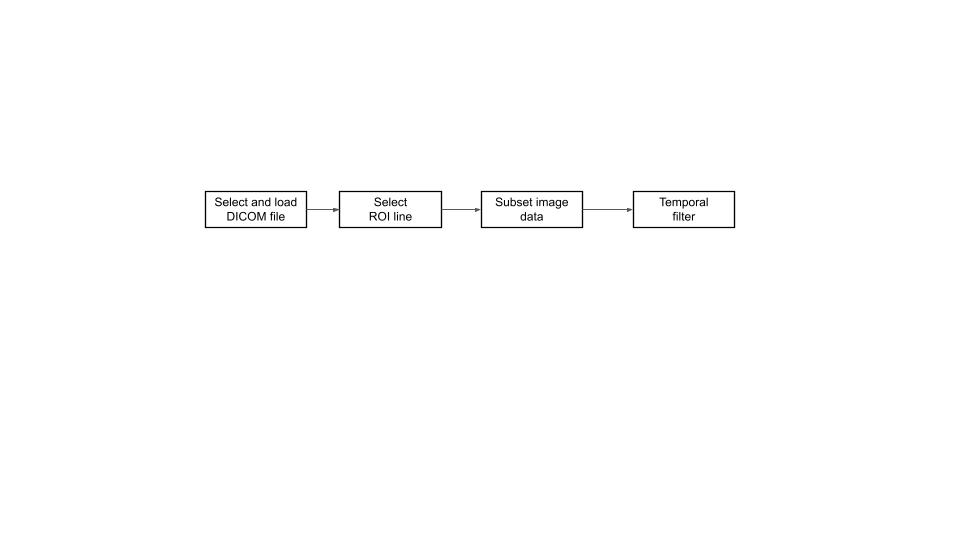


Supplemental Figure S2: Load image data and preprocess steps


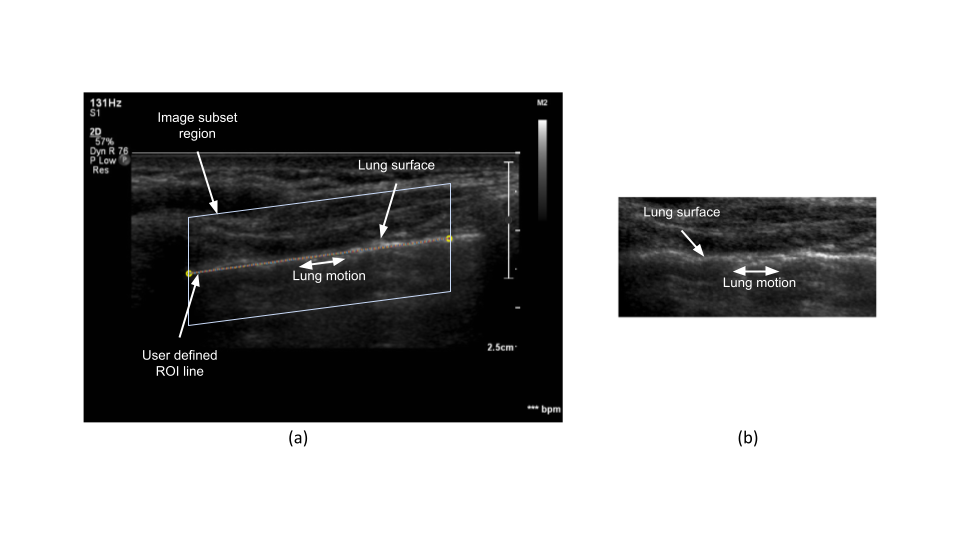


Supplemental Figure S3: Image subset example. (a) User defines a line approximately overlaying the lung surface shown in the B-mode images. The software extracts an image subset from each frame about the ROI line, illustrated by the parallelogram. (b) Sub-image created from image extraction. Lung motion is approximately 1-D (along x) in the sub-images.

## *Load image loop and preprocess*

Figure S2 shows steps for data extraction and preprocessing of DICOM B-mode image data. After the DICOM file is selected and loaded, the image data is converted from color data for each pixel (e.g., red, green, blue values) to grayscale by averaging the color channels of the image data. The subset image data is defined by the user line selection, identifying the approximate location of the lung surface, as illustrated in Figure S3. In Figure S3a, the user defined ROI line overlays the lung surface. The vertical center of the subset region is defined by the line. An equal number of pixels are included above and below each pixel of the line to define the vertical extent of the sub-image, typically 40-100 pixels depending on DICOM image pixel size. The lateral extent of the image subset is determined by the y-dimension length of the line. These steps define a parallelogram with a center line defined by the ROI line as shown in the figure. The image subset region is the same for each frame. The image sub-setting reduces the number of pixels processed in subsequent steps of the method, improving computation time and reducing memory footprint, but also makes the tracking approximately one dimensional. An example subset image is shown in the right panel of Figure S3. The lung surface motion is almost entirely along the x dimension. Reducing the dimensionality required by the tracking algorithm significantly improves robustness to noise and improves accuracy. After sub-setting, the data is high pass temporal filtered using a finite impulse response (FIR) filter. This reduces the strength of specular reflections from the lung surface as well as stationary artifacts and reverberations. This is similar to a wall or clutter filter used in Doppler and color flow imaging. Note that this operation is not as effective on B-mode data compared to radio-frequency (RF) data, since the intensity detection process to create B-mode images combines moving and stationary signals. However, it does offer improvement in reducing stationary and slow-moving image components which can degrade tracking quality.


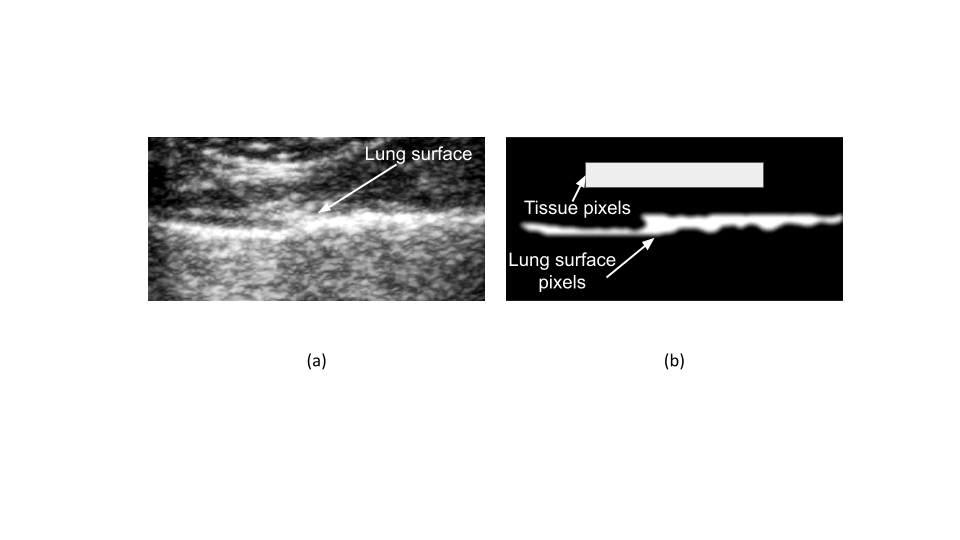


Figure S4: Example of lung surface detection and segmentation. (a) an example sub-image showing lung surface. (b) corresponding mask image representing detected pixels of the lung surface and surrounding tissue.

## *Detect lung surface and tissue region*

Each frame of the subset image stack is processed to identify pixels of the lung surface. The segmentation algorithm analyzes each column of each image and identifies the lung surface based on the B-mode strength compared to surrounding tissue. The method assumes that reflections from the lung surface are typically stronger than those from nearby tissue. Pixel values that are higher than a fraction of the maximum value of each column are identified as lung surface pixels. If the maximum pixel value of a column is below a threshold, the entire column is considered not part of the lung surface. A mask image is created for each sub-image frame, with lung surface pixels set to one and other pixel values are zero. A low pass FIR spatial filter (e.g., 5 x 5 pixel Hanning window) is applied to each mask frame and a second threshold step is applied, setting pixels with values below 0.5 to zero. This step smooths edges of the mask, reduces noise and reduces false detection regions that are small and isolated from larger regions of detected lung surface. An example B-mode sub-image is shown in Figure S4(a). The lung surface spans the image horizontally, at approximately the vertical center. The resulting lung surface mask image is presented in Figure S4(b).

Tissue near the lung surface is also identified and subsequently motion tracked to compensate for bulk tissue or probe motion. The surrounding tissue region’s vertical extent is defined as the middle one third of the upper sub-image half, and the horizontal extent is the middle half of the sub-image width, as shown in the right panel of Figure S4. The tissue region is defined to provide margin from the moving lung surface, edges of the image and more stationary issues near the body surface. Ideally the motion of the tissue region reflects any bulk tissue motion (e.g., from patient muscle movements) or probe motion during data acquisition.

Although shown as a combined image in Figure S4(b), the mask sets for the lung surface and tissue pixels can be accessed independently by the software. These masks are used to select pixels that will be used for motion tracking.


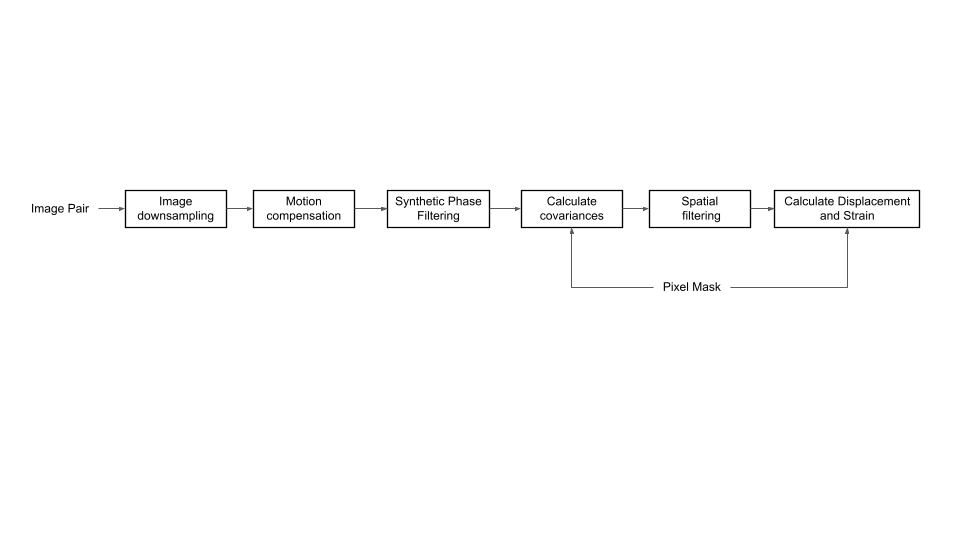


Figure S5: System for producing inter-frame displacement and strain

## *Calculate inter-frame displacements*

The core component of our method is the calculation of inter-frame displacements using synthetic phase (SP), presented in Figure S5. There are several advantages to this approach compared to the more common block and pattern matching methods for measuring tissue motion and deformation. SP displacement measurements are computationally more efficient, as tissue motion measurements between frames with sub-pixel precision are calculated from a single image cross covariance and autocovariance measurement for a given pixel. Additionally, the tracking range (the size of the tracking search region) is determined by the synthetic frequency, set by the center frequency of the SP FIR filter. In contrast, block or pattern matching speckle tracking methods (e.g., cross correlation) require the number of lags of the image cross correlation to increase with tracking range. To produce sub-pixel accuracy displacement measurements, these techniques need objective function interpolation (e.g., correlation function interpolation), image up-sampling with additional cross correlation operations, or phase zero crossing processing if the image data has phase information (e.g., axial tracking of RF data). Finally, lag based matching algorithms can suffer from noise induced peak hopping (i.e., the incorrect selection of best match lag), which can result in large displacement measurement errors. SP methods do not suffer from peak hopping, and the displacement errors are better behaved without multi-pixel jumps associated with correlation peak errors.

Figure S5 shows an overview of the SP system for measuring tissue displacement. The system performs tracking in one dimension. Unlike other speckle tracking applications (e.g. cardiac strain imaging), our tracking problem is essentially one dimensional because the image segmentation phase aligns the lung motion along the x-dimension of the sub-images. The input data is a sub-image pair that is downsampled by applying a low pass moving average anti-aliasing filter followed by spatial decimation. The sub-image pair is sequential sub-images selected from the time series of sub-images (i.e., sub-image from frame N and frame N+1) created from the image segmentation process described above. The next stage, motion compensation, is optional, and is done by resampling one of the input images based on a priori or previously calculated displacement information. The resampling spatially aligns the image pair based on displacement data using bi-linear interpolation. This can be useful when doing successive displacement measurements on image data with different resolutions (i.e., spatial downsampling).
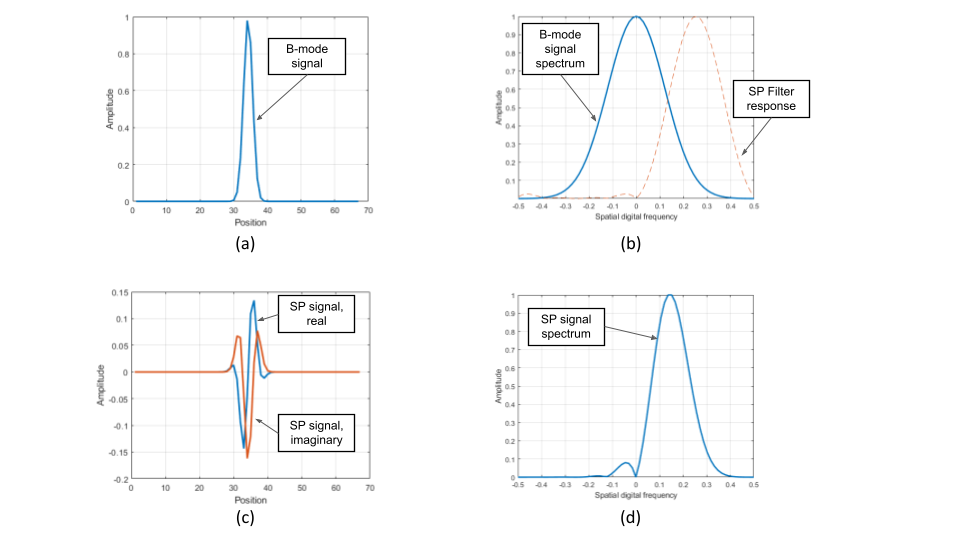


Figure S6: System for producing inter-frame displacement and strain

Synthetic phase filtering stage of Figure S5 changes the B-mode intensity image to a complex (real and imaginary components) image in which structures, such as speckle, have a phase signal that is proportional to position. An example of the SP filtering process is shown in Figure S6. The top left panel shows a Gaussian envelope that represents a line from a B-mode image passing through a speckle structure. The x-axis and y-axis are position and amplitude, respectively. The spatial spectrum of the Gaussian signal is shown by the blue curve in Figure S6(b). The x-axis is digital spatial frequency with Nyquist frequency = 0.5, and DC is at the center of the x-axis. The y-axis is the spectrum amplitude. In this case the spectrum maximum is normalized to one. The dashed red line shown in the panel represents the complex FIR filter spectral response used to create the SP data. In this example, the FIR filter is a 7 point Hanning filter centered at 0.25 digital frequency. Effectively, the filter applies a practical approximation to a Hilbert transform. The complex SP signal created after filtering is plotted in the lower left panel and the corresponding spectrum in the lower right panel. Like the B-mode spectrum, the SP spectrum maximum is set to 1. The complex SP signals are analogous to complex RF data commonly used for axial (i.e., along beam) speckle tracking and Doppler processing. Note that the cost for this approach is reduced bandwidth of the SP signals compared to unfiltered B-mode signals.

Inter-frame displacement is calculated from successive frames (i.e., frame pair) by measuring the phase change of the SP signals between frames. This is done on a per pixel basis to create a displacement image and these images are further processed to produce a lung surface displacement and strain measurement for each frame pair.

The expression for a lung speckle signal in frame 1 acquired at time t1 can be written as:

$$s(x,y,t1)=G(x-x_{t1},y-y_{t1})e^{jk(x-x_{t1})}$$

where *G* is the envelope of the SP pulse (i.e., amplitude of the speckle or tissue structure created from filtering the B-mode image), and *x* and *y* are image spatial coordinates. The location of the signal is *x_t1_*, *y_t1_*. SP filtering has been performed along the *x* dimension, resulting the *k(x-x_1_)* phase term where *k* is the wavenumber *2𝛑/𝜆*. Lambda is set by the center frequency of the SP spectrum. Similarly, the signal of frame 2 acquired at time *t2* is:

$$s(x,y,t2)=G(x-x_{t2},y-y_{t2})e^{jk(x-x_{t2})}$$

where is *x_t2_, y_t2_* is the new location of the SP pulse caused by tissue motion. In the case of constant tissue velocity, and consequently, pulse velocity between frames:

$$x_{t2}=x_{t1}+v_{x}*\Delta t$$

$$y_{t2}=y_{t1}+v_{y}*\Delta t$$

where the time between frames is $\Delta t=t2-t1$.

Our goal is to determine the change in position of the lung surface along the x dimension, *x_t2_ - x_t1_*. This is done by measuring the phase of the complex conjugate multiply (CCM) of the SP signals:

$${CCM}_{inter}\left( x,y \right)=s(x,y,t1)*conj(s(x,y,t2))=G(x-x_{t1},y-y_{t1})G(x-x_{t2},y-y_{t2})e^{j\phi_{inter}}$$

where the term of interest, the phase, is

$\phi_{inter}=k(x_{t2}-x_{t1})= k\Delta x$.

The inter subscript denotes the inter-frame CCM. The displacement for each pixel in the base image, image 1 in this example, is calculated from weighted average of the CCM over a small image chip, or kernel:

$$\sigma_{inter}(x,y,t1)=\sum_{i,j=-L/2}^{L/2} {W(x',y',t1)CCM}_{inter}(x',y',t1)$$

where *x’=x-i* and *y’=y-j*. This is the covariance of the SP signals within the kernel. The process sums the phasors over an image kernel of size L x L, with each phasor weighted by the product of the pulse amplitudes and weighting function *W*. The pixel mask images, calculated during segmentation processing described above, are used for the weighting function, as indicated by the pixel mask input to the covariance calculation shown in Figure S5. The mask pixels corresponding to the kernel position and size within the sub-image, apodized by L x L pixel Hanning window, form the covariance weighting. The weighting allows only the pixels of interest (e.g., the lung surface) to be tracked. This is particularly important for lung surface tracking because the adjacent tissue has very different motion characteristics and can corrupt the lung tissue displacement measurements if included in the covariance calculation.

To determine the displacement from the phase the wavenumber k, or equivalently the center frequency of the SP signals, is measured. To accomplish this, the CCM of the base image 1 with a one pixel shifted version of itself (i.e., lag=1 CCM) is calculated

$${CCM}_{intra}\left( x,y,t1 \right)=s\left( x,y,t1 \right)*conj\left( s\left( x-1,y,t1 \right) \right)$$

$=G(x-x_{t1},y-y_{t1})G(x-x_{t1}-1,y-y_{t1})e^{j\phi_{intra}}$ .

In this case the phase is

$\phi_{intra}=k$.

Averaging of the phasors of the intra-frame CCM is done across the kernel in the same fashion as described above for the inter-frame covariance calculation:

$\sigma_{intra}(x,y,t1)=\sum_{i,j=-L/2}^{L/2} {W(x',y',t1)CCM}_{intra}(x',y',t1)$.

This is the first lag of the autocovariance of the base image calculated over the tracking kernel.

The inter-frame and intra-frame covariances are calculated for every pixel and all successive frame pairs of the sub-image time series. As indicated by the spatial filtering block of Figure S5, the complex covariance images are spatially low pass filtered using a 2D FIR filter to reduce noise.

Next, inter-frame displacement images are calculated from the phase of the inter-frame covariance:

${\theta_{inter}(x,y,tn)=angle(\sigma'}_{A,B}(x,y,tn)) =E[k\Delta x]$,

and the phase of the intra-frame covariance:

$\theta_{intra}(x,y,tn)={angle(\sigma'}_{A_{0},A_{1}}(x,y,tn)) = E[k]$,

where *tn* is the time of the nth frame and $\sigma^{'}$ indicates the spatially filtered covariance. The E operator represents the two statistical averaging processes: weighted average of the CCM values over the kernel and spatially averaging (filtering) of the complex covariance values prior to calculation of the phase angle.

Finally, the inter-frame displacement for each pixel and frame is:

$u(x,y,tn)=\frac{\theta_{inter}(x,y,tn)}{\theta_{intra}(x,y,tn)}=\frac{E[k\Delta x]}{E[k]}$.
.

Note that lung surface velocity, $v_{x}(x,y,tn)$, is

$v_{x}(x,y,tn)=\frac{u(x,y,tn)}{\Delta t}$ .

A single inter-frame displacement measurement for each frame is calculated from the displacement images *u(x,y,tn)*. The frame displacement is the mask weighted spatial average of the displacement image:

$d(tn)=\frac{\sum_{x=1}^{J} \sum_{y=1}^{K} u(x,y,tn)M(x,y,tn)}{\sum_{x=1}^{J} \sum_{y=1}^{K} M(x,y,tn)}$ ,

where *M(x,y,tn)* is the pixel mask for frame *tn*, which identifies the lung surface or tissue region pixels, depending which region is being tracked. The total image pixels along the x and y dimensions are denoted *J* and *K*, respectively. Strain is calculated from the displacement of the right and left image halves:

$d_{L}(tn)=\frac{\sum_{x=1}^{J/2} \sum_{y=1}^{K} u(x,y,tn)M(x,y,tn)}{\sum_{x=1}^{J/2} \sum_{y=1}^{K} M(x,y,tn)}$

and

$d_{R}(tn)=\frac{\sum_{x=J/2}^{J} \sum_{y=1}^{K} u(x,y,tn)M(x,y,tn)}{\sum_{x=J/2}^{J} \sum_{y=1}^{K} M(x,y,tn)}$ ,

where *R* and *L* indicate the right and left image halves, respectively. The strain measurement for frame m is the difference in right and left image displacement divided by the separation between the displacement measurements:

$s(tn)= \frac{d_{L}(tn)-d_{R}(tn)}{\Delta_{LR}}$.

Because of the mask weighting, the spatial distribution of pixels included in the displacement calculation may not be spatially uniform. That is, the separation distance, $\Delta_{LR}$, is not necessarily *J/2* (half x-dimension length). The effective separation distance is determined from the center of mass of the mask in each image half:

$x_{R center}=\frac{\sum_{x=J/2}^{J} \sum_{y=1}^{K} xM(x,y,tn)}{\sum_{x=J/2}^{J} \sum_{y=1}^{K} M(x,y,tn)}$

and

$x_{L center}=\frac{\sum_{x=1}^{J/2} \sum_{y=1}^{K} xM(x,y,tn)}{\sum_{x=1}^{J/2} \sum_{y=1}^{K} M(x,y,tn)}$.

Therefore, the interframe strain is:

$s(tn)= \frac{d_{L}(tn)-d_{R}(tn)}{x_{R center}-x_{L center}}$.

## *Calculate accumulated displacements*

The time (frame) accumulated strain and displacement are of clinical interest, describing the overall motion and expansion and relaxation of the lung surface. The accumulated displacement and strain are given by:

$D(tn) = \sum_{m=1}^{tn} d(m)$

and

$S(tn) = \sum_{m=1}^{tn} s(m)$,

respectively

An example lung surface strain and displacement measurement calculated from a B-mode CINE loop acquired from a healthy volunteer is shown in Figure S7.


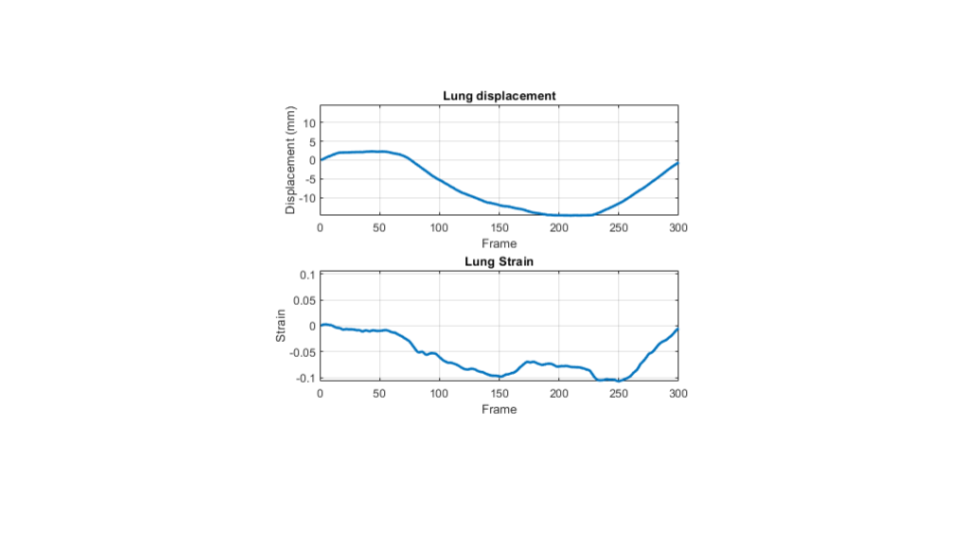


Figure S7: Example accumulated displacement and strain measurement for single breath cycle from a healthy volunteer.

The peak-to-peak displacement and strain values over the measured breath cycle are 17.5 mm and 0.1 (i.e., 10% strain), respectively. The B-mode frame rate in this example is 131 Hz, resulting in a record length of ~2.3 seconds. Note that the time record was selected by the SW operator using the mouse to define the start and end frames of a breath cycle. Only the selected cycle is shown in Figure S7. Speckle tracking drift compensation was applied to both plots by subtracting the temporal linear function that passes through the start and end frames of the breath cycle at the raw (pre-compensated) strain or displacement values. Drift compensation corrects for accumulating, non-zero mean, tracking errors (Khan et al. 2021 DOI: 10.1007/s10554-021-02200-8) and ensures the measurements at start and end of the breath cycle are identical, which is the expected behavior of cyclical, regular breath motion.

## *Tracking system application notes*

The system and methods described above produce displacement and strain for each frame of a user selected breath. The measurements may be done for multiple breaths of a given CINE loop. The number of available breaths (measurements) depends on the respiration rate and the loop length. In practice the algorithm is executed twice: to track the lung surface and also the surrounding tissue. The method for these is identical, with only the pixel mask modified to switch the target tissue. Additionally, each execution tracks x and y motion independently even though the majority of the lung surface motion is in the x dimension. The description above is focused on tracking in the x-dimension. Changing tracking dimension is done by changing the SP filter direction and inter-frame covariance lag direction.

The tracking algorithm capture range (i.e., maximum inter-frame displacement that can be measured) can be increased by reducing the center frequency of the SP filter. This is equivalent to lowering the RF carrier frequency to increase the aliasing limit. Additionally, greater image downsampling, done as part of the system of Figure S5, can be used to increase capture range.

The method supports iterative execution to improve or customize performance based on the lung motion and acquisition characteristics. For example, the algorithm can be initially run with a large image downsampling to increase capture range, followed by execution using a smaller downsampling to improve accuracy. The inter-frame displacement measurements from the coarse sampling are used for motion compensation in the higher precision second stage processing.

***Validation using simulated data***


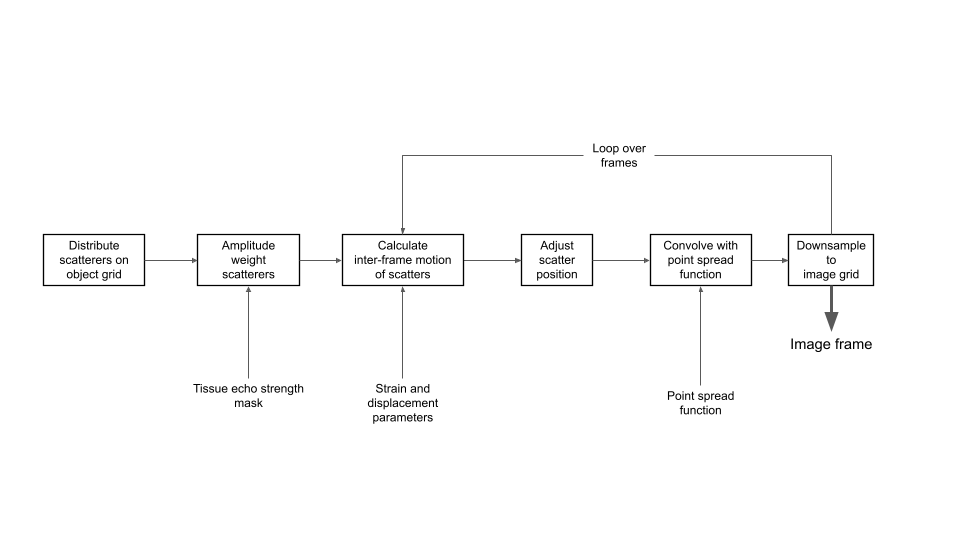


Figure S8: Method for creating simulated lung ultrasound images.

The accuracy and robustness of the tracking system was quantified using simulated ultrasound B-mode image data representing lung surface motion and deformation. The simulated data was created using Matlab. A block diagram of the method is shown in Figure S8. The object underlying the simulated image set is created by randomly distributing scatters on a spatial object grid. The object grid sampling is 8x finer than the output image sampling and the scattering density is 120 scatters per square wavelength. The scatterers are then weighted based on the desired acoustic backscatter strength, referred to as the tissue echo strength mask in Figure S8. For example, a horizontal strip of scatterers can be given high amplitude weighting to represent the scattering from the lung surface. The method allows for the combination of multiple objects which can be used to create regions of different tissue motion parameters (i.e., strain and displacement). The scatterer sets are combined for all object layers to form the underlying scatter distribution for image formation. The desired strain and displacement for each object is defined in the software. These values determine the inter-frame motion of each scatter. As indicated by Figure S8, the position of each scatter is adjusted based on the tissue dynamics parameters followed by convolution of the scatter object with a simulated point spread function to create an ultrasound image. The point spread function (PSF) is created by simulating the ultrasound image from a single point scatter. The imaging system parameters determine the characteristics of the PSF. For the simulation results presented here, a 64 element transducer with half-wavelength pitch was simulated with a center frequency and fractional bandwidth of 5 MHz and 60%, respectively. Figure S9 displays the PSF that is convolved with the object scatterers. The dark and light regions indicate high and low pressure regions of the ultrasound pulse. Note that this method uses a spatially invariant PSF which corresponds to an imaging system with dynamic transmit and receive focusing with a fixed F/#.


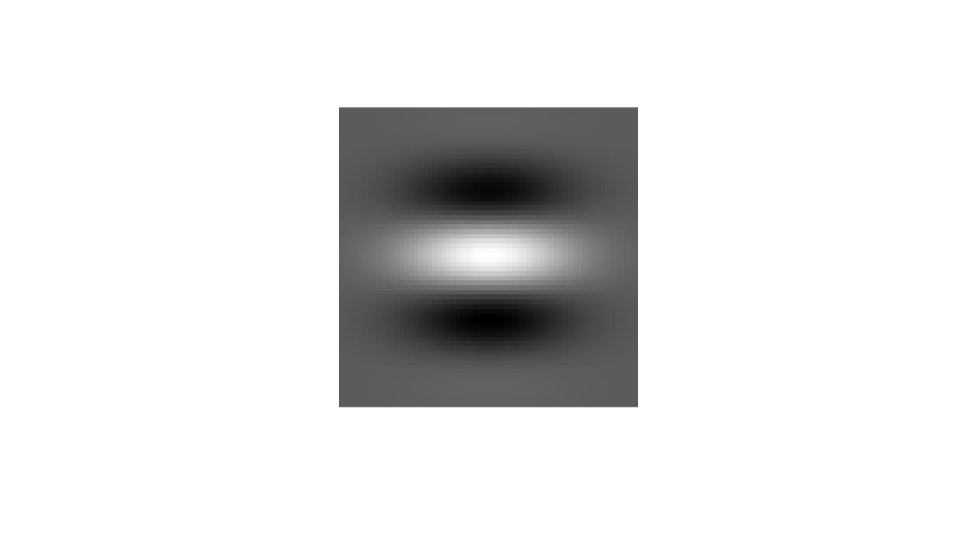


Figure S9: Simulated point spread function for convolving with object scatter distribution to create upsampled ultrasound images.

The final step in the process is downsampling the PSF convolved scatter objects to create a radio frequency (RF) ultrasound image. The downsampling factor is 8x in this case. The RF image frame is basebanded and magnitude detected to create a B-mode frame. The process from calculating inter-frame motion to downsampling is repeated for all frames as indicated by Figure S8.


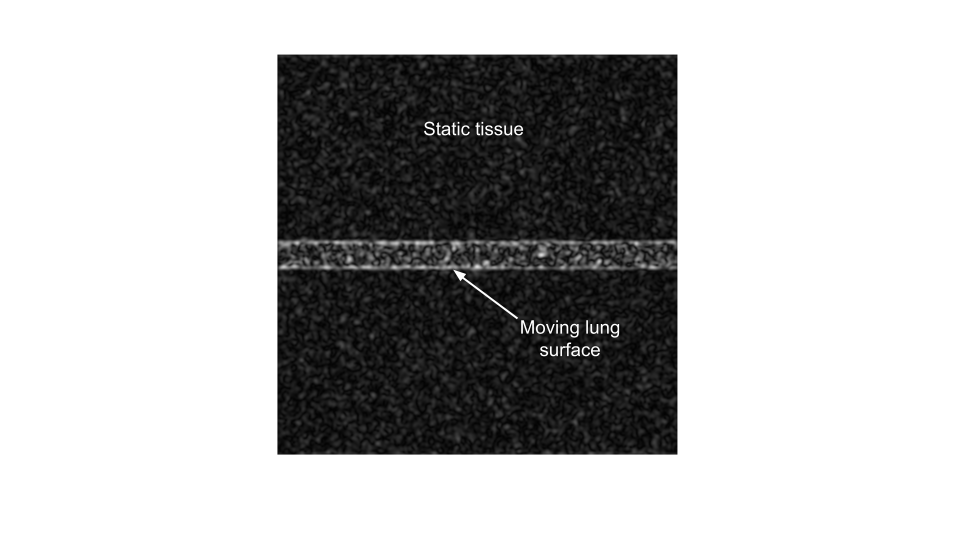


Figure S10: Simulated B-mode image of lung surface and background speckle from stationary tissue.

An example B-mode image generated from the simulated RF data is shown in Figure S10. The image is 400 x 400 pixels. Two scatterer objects were used to create the image. One set of scatterers generated the lung surface, represented by the horizontal strip in the center of the image. The scatters for the lung surface have both strain and displacement parameters assigned to it to represent lung motion. The second scatter object creates the background speckle, which is lower intensity than the lung surface, set by the tissue echo strength mask. The background speckle is static and has no motion or strain.
